# Fetal programming effects of pentaerythritol tetranitrate in a rat model of superimposed preeclampsia

**DOI:** 10.1007/s00109-020-01949-0

**Published:** 2020-08-03

**Authors:** Andy W. C. Man, Min Chen, Yawen Zhou, Zhixiong Wu, Gisela Reifenberg, Andreas Daiber, Thomas Münzel, Ning Xia, Huige Li

**Affiliations:** 1 Department of Pharmacology, Johannes Gutenberg University Medical Center, Langenbeck Str. 1, 55131 Mainz, Germany; 2 Department of Anaesthesiology, Institute of Anaesthesiology and Critical Care Medicine, Union Hospital, Tongji Medical College Huazhong University of Science and Technology, Wuhan, China; 3 Department of Cardiology I, Laboratory of Molecular Cardiology, Johannes Gutenberg University Medical Center, Mainz, Germany; 4 German Center for Cardiovascular Research (DZHK), Partner Site Rhine-Main, Mainz, Germany

**Keywords:** Fetal programming, Epigenetics, Dahl salt-sensitive rats, Pentaerythritol tetranitrate, Vascular function

## Abstract

**Abstract:**

Preeclampsia is a common medical condition during pregnancy and a major cause of maternal and prenatal mortality. The present study was conducted to investigate the effects of maternal treatment with pentaerythritol tetranitrate (PETN) in Dahl salt-sensitive rats (DSSR), a model of superimposed preeclampsia. F0 parental DSSR were treated with PETN (50 mg/kg) from the time point of mating to the end of lactation. Maternal PETN treatment improved fetal growth and had no effect on blood pressure in DSSR offspring fed with normal chow or high-salt diet. Upon high-fat diet (HFD) feeding, offspring from PETN-treated mother showed improved glucose tolerance despite similar weight gain. Unexpectedly, maternal PETN treatment significantly potentiated the HFD-induced blood pressure elevation in male DSSR offspring. Endothelium-derived hyperpolarization factor (EDHF)-mediated vasodilation was similar between NCD-fed and HFD-fed control offspring but was markedly reduced in HFD-fed PETN offspring. EDHF genes were downregulated in the vasculature of HFD-fed PETN offspring, which was associated with epigenetic changes in histone modifications. In conclusion, maternal PETN treatment in DSSR shows both beneficial and unfavorable effects. It improves fetal growth and ameliorates glucose tolerance in the offspring. Although maternal PETN treatment has no effect on blood pressure in offspring fed with normal chow or high-salt diet, the offspring is at higher risk to develop HFD-induced hypertension. PETN may potentiate the blood pressure response to HFD by epigenetic modifications of EDHF genes.

**Key messages:**

*The core findings of this article suggest that maternal PETN treatment of DSSR, a rat model of a spontaneous superimposed preeclampsia, leads to*

• *Improvement of fetal growth;*

• *No changes of maternal blood pressure or markers of preeclampsia;*

• *Amelioration of HFD-induced glucose intolerance in adult offspring;*

• *No changes in blood pressure development of the offspring on normal chow or high salt-diet;*

• *Potentiation of blood pressure elevation of the offspring on HFD.*

**Electronic supplementary material:**

The online version of this article (10.1007/s00109-020-01949-0) contains supplementary material, which is available to authorized users.

## Introduction

Gestational hypertension is one of the most common pregnancy complications and leads to 20–25% of prenatal mortality in Europe [1]. Preeclampsia refers to pregnancy-specific disorder characterized by hypertension, proteinuria occurring after the 20th completed week of pregnancy [2, 3]. Preeclamptic patients are at an increased risk of developing cardiovascular diseases and dementia later in life [4]. Fetal growth restriction refers to poor fetus growth and is usually associated with preeclampsia. Almost 10% of all pregnancies experience malperfusion of the placenta resulting in intrauterine growth restriction [5]. Fetal growth restriction may limit the growth potential of fetus and result in later adult abnormality [6, 7]. Moreover, fetal growth restriction leads to adverse molecular and physiological adaptive changes [8]. A recent genome-wide study revealed possible early epigenetic disruptions in growth-restricted or preeclamptic offspring [9], suggesting a higher risk of cardiovascular and metabolic diseases later in adulthood [8, 10].

The mechanisms of the pathology of preeclampsia remain unclear and there is currently no effective treatment available. Several studies have examined the possibility of prophylactic use of low-dose aspirin, while it appears to be inefficient in patients already developing preeclampsia [11]. In addition, aspirin may cause intracranial hemorrhage in premature infants [12]. Therefore, it is urgently needed to develop new potential treatments for preeclampsia and fetal growth restriction that are safe and beneficial for both mother and infant.

Nitric oxide (NO) donors have been shown to improve blood flow in the fetoplacental circulation of pregnancies affected by mild preeclampsia [13]. Pentaerythritol tetranitrate (PETN) possesses both NO-stimulating and antioxidant properties [14, 15]. So far, no adverse effects of maternal PETN treatment on either mother or offspring development were observed in animal or human studies [16–18]. Our group has previously demonstrated that maternal treatment of spontaneously hypertensive rats (SHR) with PETN resulted in a persistent blood pressure reduction and improvement in endothelial function through epigenetic mechanisms in the offspring [19, 20]. In a recent prospective randomized controlled study, PETN has demonstrated a beneficial effect on reducing risk of development of intrauterine growth restriction in patients at mid gestation [18]. Although there was no difference in the risk of developing preeclampsia, the early onset of preeclampsia was reduced in trend in the high-risk group by PETN. Patients receiving PETN had a significantly reduced risk of preterm birth less than 32 weeks and reduced placental abruption compared with placebo control [18]. However, whether PETN is effective in targeting preeclampsia and the effect on offspring development remain unclear.

Dahl sat sensitive rats (DSSR) are considered a genetic model of salt hypertension [21]. Renal injury and insulin resistance are also observed in DSSR [21]. Recently, a few studies have described a placental insufficiency in pregnant DSSR and considered DSSR a spontaneous superimposed preeclampsia model [22, 23]. DSSR display phenotypes that are consistent with many characteristics observed in preeclampsia patients, including proteinuria, hypertension, reduced fetal growth, and litter size [22, 23]. Moreover, since obesity is a major risk factor for hypertension [24] and high-fat diet (HFD) feeding has demonstrated controversial actions in DSSR, we fed HFD to both control and PETN offspring to examine the effect of maternal treatment on the onset of obesity-induced hypertension in DSSR. In the present study, we want to investigate the effects of maternal PETN treatment in DSSR on preeclampsia phenotypes and the effect on HFD-induced complications in adult DSSR offspring.

## Method

### Animal model

DSSR were from Charles River Laboratories (Sulzfeld, Germany). PETN-lactose (18% PETN with 82% D-lactose monohydrate) was added into normal chow (ssniff GmbH, Soest, Germany) at a concentration of 5.5 g/kg (≈ 1 g PETN /kg chow) [19]. F0 parental DSSR were fed with food ad libitum, either normal chow (control) or PETN-containing chow, from mating (at the age of 3 months) to the end of lactation period, resulting in a PETN daily dose of approximately 50 mg/kg [19]. Pregnancy was confirmed by checking the plug. Only the first birth of each breeding pair was used in the study. For high-salt diet (HSD) experiment, the F1 offspring rats from all groups were firstly fed with low-salt diet (LSD: 0.369% NaCl; E050, ssniff®, Soest, Germany) from the age of 7 weeks and then challenged with HSD (8% NaCl; E052, ssniff®, Soest, Germany) starting at the age of 8 weeks. For HFD experiment, the F1 offspring rats from all groups received either normal control diet (NCD) or high-fat diet (HFD) (HFD: 45% kcal from fat; E15744–34, ssniff®, Soest, Germany) starting at the age of 5 weeks. HFD was given for 11 weeks. Six control and six PETN breading pairs were used to study the fetal growth. Additional five control and five PETN breading pairs were used to give birth for studying the effects in adult F1 offspring. Isoflurane and intraperitoneal injection of pentobarbital were used for euthanasia. Urine samples were collected directly from the bladder after euthanasia. Urine protein was measured by bicinchoninic acid assay and creatinine level was measured using Alinity C system (Abbott, Chicago, USA). All the experiments performed involved offspring from at least three different litters. The animal experiment was approved by the responsible regulatory authority (Landesuntersuchungsamt Rheinland-Pfalz; 23 177–07/G 16–1-038) and was conducted in accordance with the German animal protection law and the National Institutes of Health (NIH) Guide for the Care and Use of Laboratory Animals.

### Blood pressure measurement

Systolic blood pressure, diastolic blood pressure, and mean blood pressure were measured noninvasively in conscious animals by a computerized system (CODA Monitor, Kent Scientific) with a volume-pressure recording sensor and an occlusion tail-cuff. Rats were placed in individual holders. The occlusion cuff and the volume-pressure recording cuff were placed close to the base of the tail. After an adaptation period of 30 min on a 37 °C warm pad, 10 preliminary measurements were performed before actual measurement. Rats were trained for restraint for 3 consecutive days prior to the actual measurement. Results are presented as the mean of at least 15 recordings on each occasion taken. The measurements were performed at the same time of a day from 2 to 4 pm by the same investigator as done in our previous studies [19].

### Vascular function study

Second order mesenteric arteries were dissected free of adherent connective tissues and placed in cold modified Krebs-Ringer bicarbonate buffer under continuous aeration with 95% O_2_/5% CO_2_. The mesenteric arteries were cut into rings with 2–3 mm long and then suspended in the chambers of a Mulvany–Halpern wire myograph system (610 M, Danish Myo Technology A/S, Aarhus, Denmark). Isometric force was recorded by a PowerLab 4SP system (AD Instruments Inc., Colorado Springs, CO, USA). The preparations were equilibrated for 30 min at the optimal resting tensions of 2.5 mN. The viability of the endothelium was tested by the relaxation response to a single dose of acetylcholine (10^−4^ M) after obtaining a reference contraction to 60 mM potassium chloride (KCl) twice prior to the actual experiment. For actual experiment, preparations were incubated for 30 min with or without different inhibitors (either 10^−4^ M *N*^G^-nitro-l-arginine, LNNA, 10^−5^ M indomethacin, indo, or both with or without 60 mM KCl). The preparations were then pre-contracted by exposing to increasing concentrations of phenylephrine (PE, 10^−9^ to 10^−5^ M). Endothelium-dependent relaxation was examined by exposure to increasing concentrations of acetylcholine (10^−9^ to 10^−4^ M). Change in tension is expressed as percentages of the PE contraction, which was adjusted to give ∼ 80% contraction of the reference KCl contraction. For calculation of the effect of drugs, area above relaxation curve (AARC) was measured in different dose-dependent curve of the preparation with the inhibitors. The difference between AARC (ΔAARC) was calculated to determine the contribution of different endothelium-dependent relaxation factors. NO-dependent relaxation was measured by the ΔAARC of control and LNNA curve. Prostaglandin (PG)-mediated relaxation was measured by the ΔAARC of control and indomethacin curve. Contribution of endothelium-dependent hyperpolarization factor (EDHF) was measured by the ΔAARC of LNNA + indomethacin and LNNA + Indo + KCl curve. The percentage contribution was calculated by referencing to the AARC of relaxation without inhibitors [19].

### Gene expression studies by quantitative PCR

Total RNA of rat thoracic aortae and mesenteric arteries were isolated using peqGOLD TriFast™ (PEQLAB) and cDNA was reverse transcribed using High-Capacity cDNA Reverse Transcription Kit (Applied Biosystems) according to previous publication [25]. QPCR was performed using SYBR Green JumpStart™ *Taq* Ready-Mix™ (Sigma-Aldrich) on an iCycler real-time PCR detection system (Bio-Rad). Quantification was achieved by the difference in quantification cycles (ΔΔCt) values that were normalized with RNA polymerase II as a reference control. Specificity of the qPCR primers were checked by melting curve analysis or gel electrophoresis of the qPCR product. The sequence of the primers used is listed in supplementary Table S1.

### Chromatin analysis

Chromatin accessibility was studied using the micrococcal nuclease (MNase, Cell Signaling Technology) digestion method. Thoracic aortae were homogenized and MNase digested for 1 h at 37 °C. The genomic DNA was then isolated and collected using ChIP DNA purification kit (Active Motif). Quantitative PCR was performed using 5 ng DNA samples as template. In general, open chromatin regions were more susceptible to MNase digestion resulting in a greater delay of quantification cycle (C_T_), while closed chromatin regions were protected from the MNase digestion resulting in minimal delays in C_T_ value, compared with that of undigested templates. The sequence of the primers used is listed in supplementary Table S2.

### Chromatin immunoprecipitation (ChIP)

Thoracic aortae were cross-linked in 1.5% formaldehyde (in PBS) at room temperature for 15 min. After adding glycine to a final concentration of 125 mM, aorta samples were homogenized in Pierce™ IP lysis buffer (Thermo Fisher Scientific) containing 1% (*v*/v) protease inhibitor cocktail and chromatin fragments of 500–1000 base pair was obtained by sonication. The lysate was incubated with either 1.5-μg-specific primary antibody or IgG at 4 °C overnight with rotation. Pierce™ Protein A agarose beads (Thermo Fisher Scientific) were added to the lysates and incubated for another hour. After washing for four times, the chromatin fragments were eluted into a TE buffer containing proteinase K. Quantitative PCR was performed using 10% volume of the DNA samples and quantified by using the Ct values for normalization against the input control, for which 1% of the DNA samples were used for qPCR [19]. H3K9 (Antibody: #17–625, Millipore) and H3K27 (Antibody: #17–622, Millipore) trimethylations are repressive epigenetic marks associated with transcriptional repression while H3K4 trimethylation (Antibody: #17–614, Millipore) is an active epigenetic marks in transcriptional active euchromatin and associated with transcriptional activation. Primers used are listed in supplementary Table 2.

### ELISA assay

Rat soluble fms-like tyrosine kinase-1 (sFlt-1, #MBS2602003) and placenta growth factor (PLFG, #MBS026910) were tested in placenta and serum with enzyme-linked immunosorbent assay (ELISA) according to the manufacturer’s instruction (Mybiosource). In brief, 1-mg protein of rat placenta (homogenized in Pierce™ IP lysis buffer containing 1% (*v*/v) protease inhibitor cocktail) and 100-μl serum were added as samples. Samples and standard were then covered and incubated at room temperature for 90 min with gentle shaking. After washing twice, biotinylated antibodies were added and incubated at room temperature for another 60 min. After washing for three times, HRP-Avidin was added and incubated for 30 min at room temperature with gentle shaking. After washing for five times, color reagent was added in dark with gentle shaking. After 30 min of incubation, stop solution was added and the absorption was read at 450 nm immediately with Sunrise™ microplate reader (Tecan Group) and analyzed by Magellan™ software (Tecan Group).

### Glucose tolerance test

Glucose tolerance test (GTT) was performed in rats after 6-h fasting at the age of 16 weeks or after overnight fasting at the age of 30 weeks. After measuring basal blood glucose levels, each rat was injected with glucose (2 g per kg body weight) intraperitoneally (i.p.). Blood glucose levels were measured 15, 30, 60, 90, and 120 min after glucose injection. Blood glucose was measured by needle puncture in the tail vein with a glucometer (Contour® XT) in conscious unrestrained rats.

### Insulin tolerance test

Insulin tolerance test (ITT) was performed in rats after 6-h fasting at 16 weeks or after 16-h overnight fasting at 30 weeks. After measuring basal blood glucose levels, each rat was injected with 0.5 U per kg body weight of insulin intraperitoneally (i.p.). Blood glucose levels were measured 15, 30, 60, 90, and 120 min after insulin injection. Blood glucose was measured by needle puncture in the tail vein with a glucometer (Contour® XT) in conscious unrestrained rats.

### Statistics

Results were expressed as mean ± SEM (standard error of the mean). If otherwise stated in the figure legends, Student’s *t* test or two-way ANOVA were used for comparison between groups. *P* values < 0.05 were considered significant. GraphPad Prism (GraphPad Software, La Jolla, CA, USA) was used to generate graphs and statistical analysis.

## Results

### Maternal PETN treatment of DSSR improves fetal growth

**Fig. 1:**
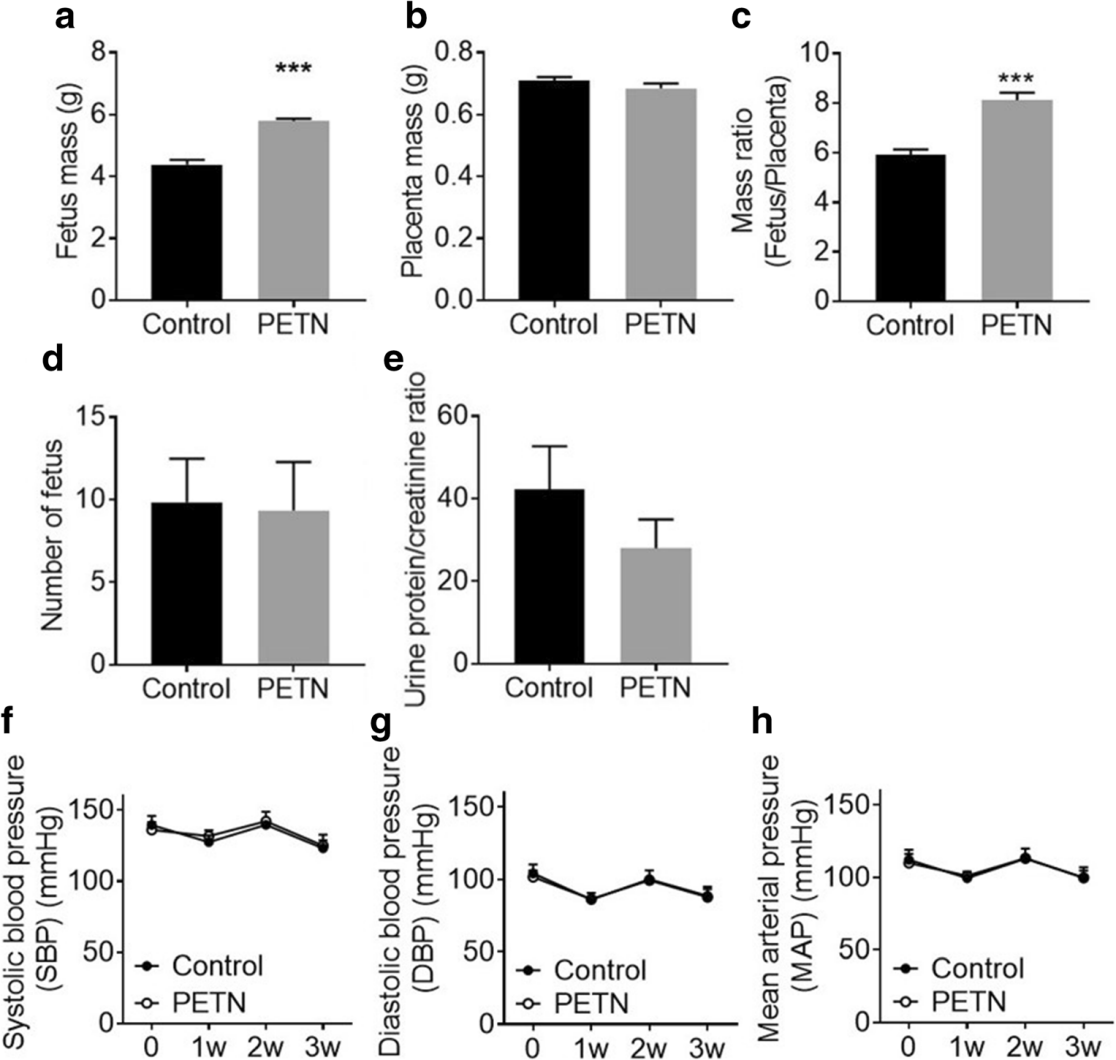
Maternal PETN treatment of DSSR improves fetal growth. F0 DSSR were treated with or without PETN (50 mg/kg/day) during pregnancy. At day 20 of pregnancy, F0 DSSR were sacrificed. The fetus mass (a), placental mass (b), fetus/placental mass ratio (c), litter size (d) were measured. (e) Urine from pregnant DSSR was collected and the protein amount was determined and calculated as the ratio to creatinine. During pregnancy, systolic blood pressure (SBP) (f), diastolic blood pressure (DBP) (g) and mean arterial pressure (MAP) (h) F0 DSSR mother was monitored weekly. Column represents mean ± SEM, *n* = 6. Student’s t test was used for comparison of PETN group with control group. ***P < 0.001 vs control group

At day 20 of pregnancy, the mass of PETN pups (5.79 ± 0.07 g) was significantly higher than control pups (4.38 ± 0.15 g), while there was no significant difference in the mass of placenta. Thus, the resulting fetus/placenta mass ratio was also significantly increased in the PETN group compared with that of control (Fig. 1 a to c). Similar results were observed in the body weights of newborn DSSR at day 1. The body weight of PETN F1 offspring (6.52 ± 0.06 g) was significantly higher compared with that of control group (6.02 ± 0.05 g) (supplementary Fig. S1).

When investigating the phenotype of preeclampsia, the litter size and protein content in urine showed no significant difference between F0 mother in the control group and PETN treatment group (Fig. 1 d and e). Pregnant DSSR showed no significant difference in blood pressure between control and PETN group during pregnancy (Fig. 1 f to h). Circulating and placental sFlt-1/PIGF ratio at late pregnancy was not affected by maternal PETN treatment (supplementary Fig. S1).

Next, we fed the offspring with either HSD or HFD. Maternal PETN treatment had no effect on either body weight change (supplementary Fig. S2) or blood pressure development (supplementary Fig. S3) in the HSD-fed offspring up to the age of 14 weeks. Therefore, we investigated the effect of maternal PETN treatment in HFD-fed DSSR F1 offspring.

### Effects of maternal PETN treatment on weight gain and metabolism in HFD-fed F1 DSSR

**Fig. 2:**
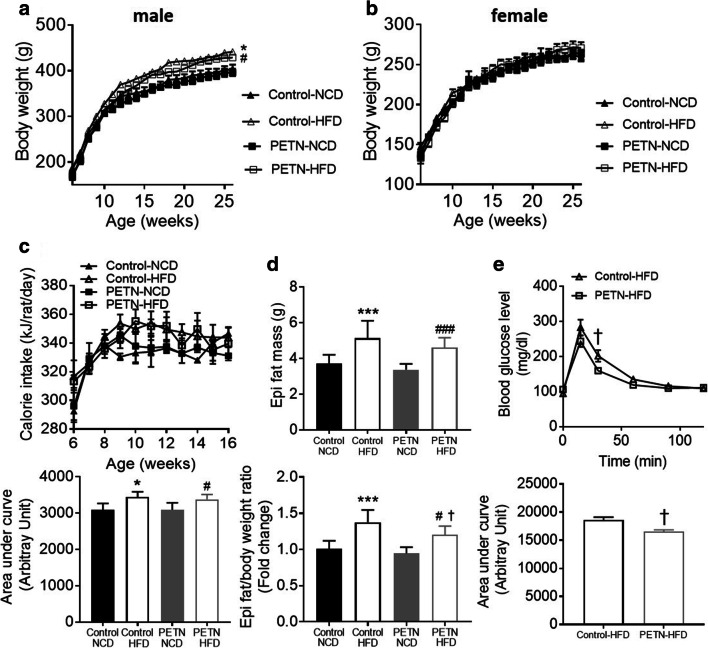
Effects of maternal PETN treatment on weight gain and metabolism in HFD-fed F1 DSSR. F0 DSSR were treated with or without PETN (50 mg/kg/day) during pregnancy and lactation periods. F1 DSSR male and female received either normal chow (NCD) or high-fat diet (HFD) (45% kcal from fat) starting at the age of 5 weeks. The weight of F1 DSSR male (a) and female (b) were monitored from 5 weeks to 25 weeks. (c) Calorie intake was monitored from male F1 DSSR from 6 weeks to 16 weeks and the area under curve (bottom) was calculated for comparison. (d) Total epididymal adipose tissue weight of 16-week-old male DSSR offspring was measured and the epididymal adipose tissue/body weight ratio was calculated for comparison (bottom). (e) Glucose tolerance test (GTT) was performed in HFD-fed 16-week-old male DSSR offspring with or without maternal PETN treatment and the area under GTT curve (bottom) was calculated for comparison. A lower area under curve represents a faster glucose metabolism. Data were presented as mean ± SEM, *n* = 3–6 for (b) *n* = 12–25 for (a), (c–e). Student’s *t* test and one-way ANOVA were used for the comparison of respective NCD and HFD groups. *P < 0.05, ***P < 0.001, vs control-NCD group. ^#^*P* < 0.05, ^###^*P* < 0.001, vs PETN-NCD group. †*P* < 0.05 vs control-HFD group

After 11 weeks of HFD feeding, the body weight of F1 male HFD-fed control and PETN DSSR was significantly higher compared with their respective NCD group (Fig. 2a). In contrast, HFD feeding showed no significant effect on body weight gain of female DSSR offspring, neither in control nor in PETN offspring (Fig. 2b). HFD feeding led to an increase in blood pressure in female F1 DSSR, with no significant effect of maternal PETN treatment on HFD-induced blood pressure elevation in the female offspring (supplementary Fig. S4). Therefore, the following experiments were only performed in male DSSR offspring.

In the male offspring, HFD-fed control and PETN group showed significant increase in calorie intake compared with their respective NCD group (Fig. 2c). In the 16-week old male DSSR offspring, the weight of epididymal adipose tissue was significantly increased in HFD-fed control and PETN group compared with their respective NCD group. Also, PETN-HFD had a significant reduction in the epididymal adipose tissue weight compared with control-HFD (Fig. 2d). GTT result suggested that there was an improved glucose tolerance in PETN-HFD compared with control-HFD (Fig. 2e), while there was no significant difference in response to insulin between the two groups (supplementary Fig. S5). The beneficial effect of maternal PETN treatment on glucose metabolism was also observed in 28-week-old PETN-HFD group (23 weeks of HFD feeding) (supplementary Fig. S6).

### Maternal PETN treatment potentiates HFD-induced blood pressure increase in DSSR

**Fig. 3:**
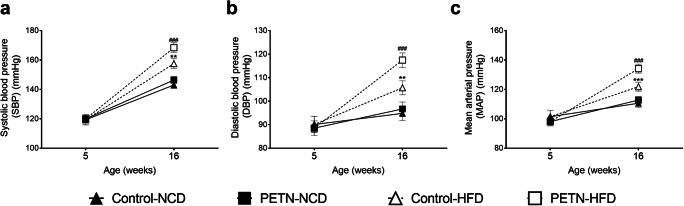
Effects of maternal PETN treatment on blood pressure in HFD-fed F1 DSSR. F0 DSSR were treated with or without PETN (50 mg/kg/day) during pregnancy and lactation periods. F1 male DSSR received either normal chow (NCD) or high-fat diet (HFD) (45% kcal from fat) starting at the age of 5 weeks. Systolic blood pressure (SBP) (a), diastolic blood pressure (DBP) (b) and mean arterial pressure (MAP) (c) were measured in the offspring at age of 5 weeks and 16 weeks. The symbols represent mean ± SEM, *n* = 10–19. Student’s *t* test was used for comparison. **P < 0.01, ***P < 0.001, vs control-NCD group. ^###^*P* < 0.001 vs PETN-NCD group

At the age of 5 weeks, there was no significant difference in the blood pressure between control and PETN groups. When the offspring were put on NCD from the age of 5 weeks to the age of 16 weeks, no difference in blood pressure was observed between control and PETN groups. HFD feeding led to an increase in blood pressure (Fig. 3 a to c). The mean arterial blood pressure of control-HFD group was ~ 10 mmHg higher than that of control-NCD group at the age of 16 weeks (Fig. 3c). HFD feeding led to a much larger blood pressure increase in PETN offspring. At the age of 16 weeks, the mean arterial blood pressure of PETN-HFD group was ~ 20 mmHg higher than that of PETN-NCD group (Fig. 3c).

### Maternal PETN treatment induces endothelial dysfunction in HFD-fed F1 DSSR

**Fig. 4:**
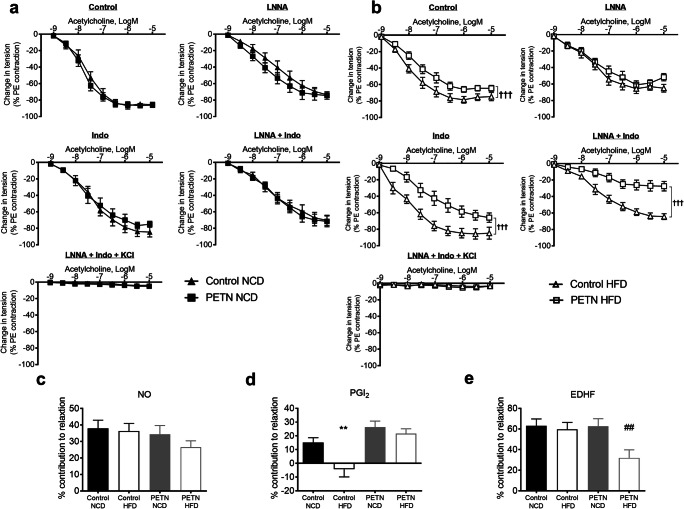
Effects of maternal PETN treatment on vascular responsiveness in HFD-fed F1 DSSR. F0 DSSR were treated with or without PETN (50 mg/kg/day) during pregnancy and lactation periods. F1 male DSSR received either normal chow (NCD) (a) or high fat diet (HFD) (45% kcal from fat) (b) starting at the age of 5 weeks. At the age of 16 weeks, F1 male DSSR was sacrificed and the second order mesenteric artery was used to measure the vascular responsiveness in a wire myograph system. Preparations were incubated for 30 min with or without of different inhibitors (either 10^−4^ M *N*^G^-nitro-l-arginine, L-NNA, 10^−5^ M indomethacin, or both with or without 60 mM KCl). The preparations were then pre-contracted by exposing increasing concentrations of phenylephrine (10^−9^ to 10^−5^ M). Endothelium-dependent relaxation was examined by exposed to increasing concentrations of acetylcholine (10^−9^ to 10^−4^ M). Change in tension is expressed as percentages of the PE contraction, which was adjusted to give ∼ 80% contraction to the reference KCl contraction. For calculation of the effect of the drugs, the area above relaxation curve (AARC) was measured in different dose-dependent curves of the preparation incubated with different inhibitors. The difference between AARC (ΔAARC) was calculated to determine the contribution of different endothelium-dependent relaxation factors compared with basal relaxation. (c) NO-dependent relaxation was measured by the ΔAARC of control and LNNA curve. (d) Prostaglandin (PG)-mediated relaxation was measured by the ΔAARC of control and indomethacin curve. (e) Contribution of endothelium-dependent hyperpolarization factor (EDHF) was measured by the ΔAARC of LNNA + indomethacin and LNNA + Indo + KCl curve. The percentage contribution was calculated by referencing to the AARC of relaxation without inhibitors. Each point represents mean ± SEM, *n* = 7–9. Student’s *t* test was used for comparison. **P < 0.01 vs control-NCD group. †††*P* < 0.001 vs control-HFD group. ^##^*P* < 0.01 vs PETN-NCD group

In NCD-fed F1 DSSR, there was no significant difference in basal endothelium-dependent vasodilator response to acetylcholine between control and PETN groups. Upon the incubation with different inhibitors, the acetylcholine-induced vasodilation was reduced, but there was no significant difference between the two groups. Similar vascular functions were observed between the NCD-fed control and PETN groups (Fig. 4a). However, a significant reduction in the basal acetylcholine-induced vasodilation was observed in PETN-HFD group compared with control-HFD. After incubation with L-NNA, there was no significant difference in the vasodilator response to acetylcholine, suggesting the vasodilator response was more NO-dependent in control-HFD. Interestingly, incubation with indomethacin resulted in a clear reduction in the vasodilation in PETN-HFD while a slightly increased acetylcholine-induced vasodilation was observed in control-HFD. On the other hand, vasodilation was minimized when the vessel was incubated with both L-NNA and indomethacin in PETN-HFD group but not control-HFD (Fig. 4b).

The contribution of different vasodilator was compared between the groups. Although PETN-HFD groups showed a trend of reduced NO-dependent relaxation, there was no significant difference between groups (Fig. 4c). Interestingly, HFD feeding significantly reduced the PG-mediated relaxation in control DSSR and prostaglandin-mediated vasoconstriction was observed. In contrast, maternal PETN treatment normalized the HFD-mediated change in the contribution of prostaglandins in relaxation (Fig. 4d). In the presence of both NO and COX inhibitor, HFD feeding resulted in a significant reduction in the EDHF-mediated relaxation in PETN-HFD but not in control-HFD (Fig. 4e).

Collectively, these results demonstrated that maternal PETN treatment normalized the HFD-induced reduction in prostaglandin-mediated relaxation, but also potentiated HFD-induced aberration of EDHF-mediated relaxation.

### Maternal PETN treatment induces epigenetic changes in blood vessel of HFD-fed F1 DSSR

**Fig. 5:**
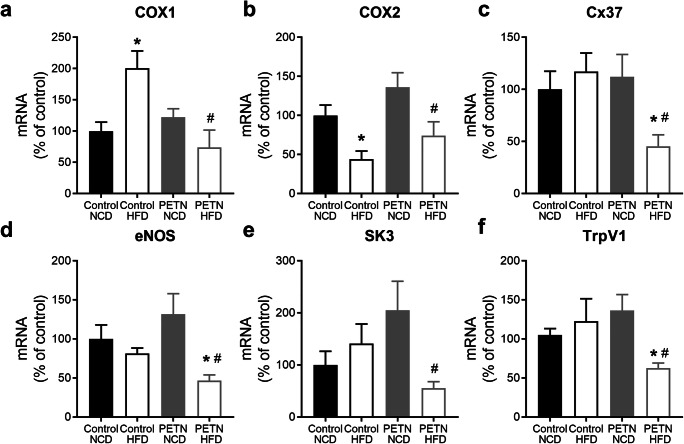
Effects of maternal PETN treatment on gene expression in HFD-fed F1 DSSR. F0 DSSR were treated with or without PETN (50 mg/kg/day) during pregnancy and lactation periods. F1 male DSSR received either normal chow (NCD) or high-fat diet (HFD) (45% kcal from fat) starting at the age of 5 weeks. Gene expressions of cyclooxygenase 1 (COX1) (a), cyclooxygenase 2 (COX2) (b), connexin-37 (Cx37) (c), endothelial nitric oxide synthase (eNOS) (d), small conductance calcium-activated potassium channel 3 (SK3) (e), and transient receptor potential cation channel subfamily V member 1 (TrpV1) (f) were measured in the aorta of 16-week male F1 DSSR with quantitative real-time PCR. Column represents mean ± SEM, *n* = 6. Student’s *t* test was used for comparison. *P < 0.05 vs control-NCD group; ^#^*P* < 0.05 PETN-NCD group

Interestingly, COX1 gene expression was significantly induced by HFD in control DSSR, while maternal PETN treatment significantly normalized such change (Fig. 5a). In contrast, COX2 gene expression was significantly downregulated by HFD in control DSSR, while it was significantly upregulated by maternal PETN treatment (Fig. 5b). HFD feeding had no significant effects on the gene expressions of eNOS, small conductance calcium-activated potassium channel 3 (SK3), connexin-37 (Cx37), and transient receptor potential cation channel subfamily V member 1 (TrpV1) in control DSSR, but these genes were significantly downregulated in PETN-HFD group (Fig. 5 c–f). Maternal PETN treatment also significantly upregulated connexin-40 (Cx40), superoxide dismutase 3 (SOD3), and transient receptor potential cation channel subfamily V member 4 (TrpV4) in NCD DSSR but these genes were downregulated in PETN-HFD group (supplementary Fig. S7). The gene expression changes were also confirmed in mesenteric arteries of the HFD-fed DSSR and similar results were obtained (supplementary Fig. S8).

**Fig. 6:**
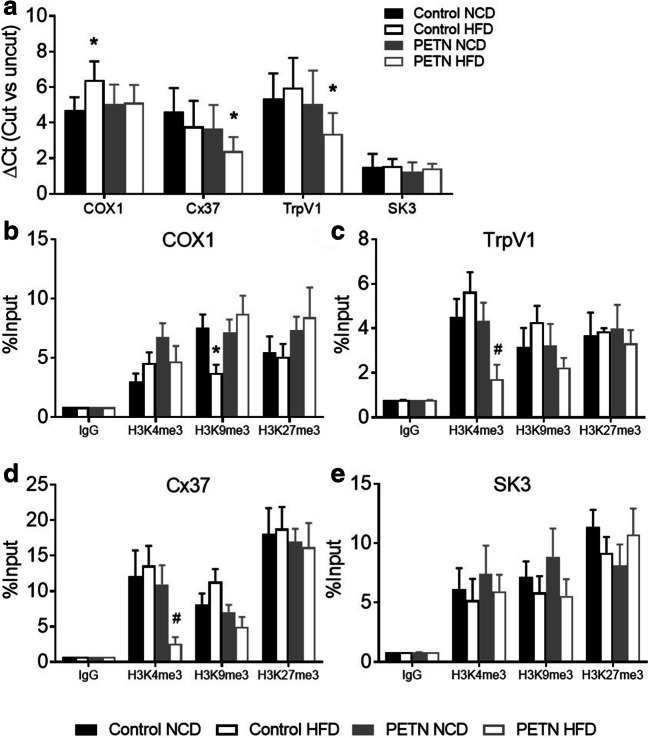
Effects of maternal PETN treatment on epigenetic regulations in HFD-fed F1 DSSR**.** (a) The chromatin accessibility at the proximal promotor regions around the transcription start site of COX1, Cx37, TrpV1, and SK3 in the aorta of 16-week male F1 DSSR was studied by MNase digestion. Open chromatin regions were more susceptible to MNase digestion resulting in a greater difference of quantification cycle (ΔC_T_), while closed chromatin regions were protected from the MNase digestion resulting in minimal change (ΔC_T_), compared with that of undigested templates. Histone 3 lysine 4 trimethylation (H3K4me3), histone 3 lysine 9 trimethylation (H3K9me3), and histone 3 lysine 27 trimethylation (H3K27me3) at the proximal promotor regions around the transcription start site of COX1 (b), TrpV1 (c), Cx37 (d), and SK3 (e) were studied with chromatin immunoprecipitation (ChIP) followed by quantitative PCR using the aorta of 16-week male F1 DSSR. Non-specific IgG was used as negative control of the ChIP experiment. Column represents mean ± SEM, *n* = 6. Student’s *t* test was used for comparison. *P < 0.05 vs control-NCD group; ^#^*P* < 0.05 PETN-NCD group

MNase digestion result showed a significant increase in the ΔCt of COX1 in control-HFD compared with control NCD. There was a significant reduction in the ΔCt of Cx37 and TrpV1 in PETN-HFD compared with PETN-NCD, whereas no significant difference was found in the accessibility of SK3 gene (Fig. 6a). These results indicated an increased chromatin accessibility in the proximal promoter regions of COX1 in control-HFD group, while there was a reduction in chromatin accessibility of Cx37 and TrpV1 gene in PETN-HFD group.

Next, histone modifications in proximal promoter regions of these genes were examined by ChIP-PCR. In the proximal promoter regions around the transcription start site of COX1, H3K9 trimethylation was significantly reduced by HFD feeding in control DSSR while maternal PETN treatment normalized the effect of HFD (Fig. 6b). This indicated that HFD feeding induced COX1 expression by epigenetic control in control DSSR which was normalized by maternal PETN treatment.

In the proximal promoter regions around the transcription start site of TrpV1 and Cx37, H3K4 trimethylation was significantly reduced in PETN-HFD compared with that of PETN-NCD (Fig. 6 c and d). The above result suggested that HFD feeding stimulated the downregulation of TrpV1 and Cx37 expression by epigenetic control in PETN group.

In coherent to MNase digestion result, H3K4, H3K9, and H3K27 trimethylations were not significantly changed in the proximal promoter regions around the transcription start site of SK3 in all groups of DSSR (Fig. 6e).

## Discussion

The present study shows that maternal PETN treatment of DSSR, a rat model of a spontaneous superimposed preeclampsia, leads to (i) an improvement of fetal growth; (ii) no changes of maternal blood pressure or markers of preeclampsia; (iii) amelioration of HFD-induced glucose intolerance in adult offspring; (iv) no changes in blood pressure development of the offspring on normal chow or high salt-diet; and (v) potentiation of blood pressure elevation in offspring on HFD.

DSSR is reported to have intrauterine growth restriction and a significantly reduced pup weight compared with normal Sprague-Dawley rat [22, 23]. Our results show that maternal PETN treatment improved the birth weight of DSSR pups (Fig. 1). Similar positive effect of maternal PETN treatment has been observed in a randomized, double-blinded trial. PETN treatment can significantly improve pregnancy outcome evidenced by the significant reduction of fetal growth restriction and perinatal death by 39% [17]. The results of present study are also consistent with those of the clinical trial by Schleussner et al. [18]. PETN treatment in patients with pre-existing risk factors showed a significant reduction in intrauterine growth restriction and preterm delivery but not the risk for preeclampsia [18]. In addition, the clinical trial was performed in women with abnormal placental perfusion at 19–24 weeks of gestation, suggesting that PETN can effectively reduce the risk of intrauterine growth restriction and preterm death. This is superior compared with low-dose aspirin, as the later seems to have no effect after 16 weeks of gestation [11]. The beneficial effect of PETN in maintaining the intrauterine growth could be attributed to the enhanced NO production and reduced oxidative stress in the placental circulation, which lead to an improvement in utero- and fetoplacental perfusion [18].

Several recent studies have shown that DSSR exhibit diet-induced blood pressure increase independent of salt intake [26–28]. HFD feeding has demonstrated controversial effects in DSSR. Some reports suggest that HFD feeding can induce hypertension in DSSR, as accompanied by body weight increase, visceral fat accumulation, and insulin resistance [26], while some suggest that HFD only exacerbated the salt-induced elevation in blood pressure and renal injury [29–31]. There are also contradictory reports showing that HFD-induced endothelial dysfunction in DSSR is through generating oxidative stress [32, 33], while others show no significant change in vascular responses of HFD-fed DSSR [34, 35]. A recent study suggests that maternal treatment with sildenafil improves fetal growth and vascular function in NCD-fed DSSR offspring [23]. How these offspring would response to HFD challenge is unknown. Our data demonstrates an exacerbation of HFD-induced hypertension in male DSSR after maternal PETN treatment. HFD-induced blood pressure elevation is exacerbated in male DSSR offspring with maternal PETN treatment while there is no further increase in body weight. Both preclinical and rat studies on fetal growth restriction suggested that hypertension development often occurs in male offspring compared with female offspring [23, 36]. Therefore, male offspring with fetal growth restriction is more prompted to the effects from prenatal interventions and diets.

Maternal PETN treatment improves glucose metabolism in DSSR, as well as reduces the weight of adipose tissue. These data further support that maternal PETN treatment can improve the metabolic phenotype of the preeclamptic offspring, probably through the prevention of early epigenetic disruptions, and result in a reduced risk of metabolic diseases later in adulthood [8, 10]. We did not investigate this in detail, because metabolism is not the focus of the study.

Recent findings suggest the potential involvement of epigenetic mechanisms in the progression of endothelial dysfunction in metabolic diseases [37, 38]. A close correspondence between gene expression and post-translational histone modifications is found in proinflammatory molecules, including tumor necrosis factor alpha (TNF-α) and COX2 under metabolic and cardiovascular diseases [38, 39]. Nevertheless, the detailed epigenetic changes that affect endothelium-dependent factors have not been studied in endothelial dysfunction in metabolic diseases. In our previous studies, maternal treatment of PETN has been shown to induce epigenetic changes and beneficial effects in aorta relaxation [19] and kidney function [20]. In the present study, we focus on studying the endothelial function of the second order mesenteric arteries in DSSR. In NCD-fed control DSSR, acetylcholine-induced vascular relaxation is reduced in the presence of indomethacin compared with basal condition due to the inhibition of COX. However, HFD feeding in DSSR shows a clear improvement of acetylcholine-induced vascular relaxation in the presence of indomethacin, as well as an increase in COX1 and a reduction in COX2 expression. COX1-derived PG may evoke endothelium-derived vasoconstrictor activity in HFD-fed control [40–42]. Interestingly, maternal PETN treatment in DSSR slightly improves the PG-mediated vascular relaxation in NCD and normalizes the deteriorate effect of HFD in PG-mediated vascular relaxation by adjusting the expression of COX1 and COX2 (Fig. 6). The epigenetic regulation of COX2 has been extensively studied [43]. However, the epigenetic changes of COX1 are less known. In the present study, we have shown that COX1 expression and the HFD-induced change in PG-mediated vasoconstrictor activity can be reversed by the epigenetic effect of maternal PETN treatment.

On the other hand, HFD feeding significantly reduces EDHF in DSSR with maternal PETN treatment but not in control DSSR. EDHF is the NO-/PG-independent component of endothelium-dependent relaxation that is prominent in the microcirculation as well as mesenteric arteries [44, 45]. Therefore, the reduction of EDHF in HFD-PETN group may have prominent contribution to the exacerbation of blood pressure elevation. To date, epigenetic control of the EDHF pathway is still poorly studied. TrpV1 is one of the essential calcium channels in endothelial cells which plays a role in EDHF-mediated response [46]. A reduced TrpV1-dependent modulation of blood flow is observed in diabetic mouse model [47]. Connexin expression is reported to be regulated by epigenetic mechanisms in various cell types [48]. Interestingly, Cx37 and Cx40 are tightly associated and affect the expression of eNOS [49]. Involvement of connexin in vasculature is critical for normal endothelial function and EDHF-mediated response. Maternal PETN treatment with HFD feeding in DSSR epigenetically changes the expression of these genes leading to a loss in EDHF-mediated relaxation. Despite of the improvement in PG-mediated vascular relaxation, the diminished EDHF-mediated response in HFD-PETN group leads to an elevation of blood pressure.

In this study, maternal PETN treatment shows a significant improvement in fetus growth and moderate effect in blood pressure control in DSSR offspring. In contrast to our previous studies [19], we notice the epigenetic effect of maternal PETN treatment in DSSR may deteriorate HFD-induced blood pressure elevation. The effect of maternal PETN treatment is dependent on models or nutrient balances during later stage of life. Therefore, it is crucial to fully investigate the targets of maternal PETN treatment in order to optimize the beneficial effect of PETN as a potential therapeutic drug. The genes involved in EDHF-mediated response are some of the important mediators that epigenetically changed by maternal PETN treatment and induced by HFD feeding. The limitation of the current study is the lack of a gene screening for all the potential candidates that are “primed” by maternal PETN treatment. Therefore, these parameters should be taken into account when considering further development of potential maternal treatments.

In conclusion, maternal PETN treatment shows improved fetal growth in a rat model of superimposed preeclampsia and had no effects on blood pressure development in offspring on normal chow or high-salt diet. In HFD-fed offspring, maternal PETN treatment was beneficial to glucose metabolism without significant effects on bodyweight gain. However, maternal PETN treatment exacerbated blood pressure elevation in HFD-fed offspring. Epigenetic changes in vasculature modulated by HFD and maternal PETN treatment contributed to the improvement of PG-dependent vascular relaxation and blunted EDHF-mediated vascular relaxation in DSSR. In this model, maternal PETN treatment leads to a plasticity of the offspring phenotype which can be affected by HFD in both beneficial and detrimental directions. Based on the above findings, it is proposed that maternal PETN treatment in DSSR may represent a novel potential treatment for preeclampsia which benefits both the mother and offspring, as evidenced by an improvement of fetal growth and amelioration of glucose intolerance of the offspring in adult age. However, the offspring should avoid high-fat diets in later life because of the potentially higher risk of blood pressure elevation in response to high-fat diet.

## Electronic supplementary material

ESM 1 (PDF 482 kb)

## Data Availability

The datasets used and/or analyzed during the current study are available from the corresponding author on reasonable request.
