# Supplementary material for: Fetal programming effects of pentaerythritol tetranitrate in a rat model of superimposed preeclampsia
Source: J Mol Med (Berl). 2020 Aug 3;98(9):1287–99. doi: 10.1007/s00109-020-01949-0 (PMC7447665; doi:10.1007/s00109-020-01949-0)
Supplement: Supplementary file 1 — (PDF 482 kb) [file 109_2020_1949_MOESM1_ESM.pdf]

Supplementary data

Fetal programming effects of pentaerythritol tetranitrate in a rat model of superimposed preeclampsia

Andy W C Man*[,1], Min Chen*[,2,1], Yawen Zhou[1], Zhixiong Wu[1], Gisela Reifenberg[1], Andreas Daiber[3,4], Thomas Münzel[3,4], Ning Xia[1], Huige Li[1]

[1]Department of Pharmacology, Johannes Gutenberg University Medical Center, Mainz, Germany

[2]Department of Anaesthesiology, Institute of Anaesthesiology and Critical Care Medicine, Union Hospital, Tongji Medical College, Huazhong University of Science and Technology, Wuhan, China

[3]Center for Cardiology, Cardiology I - Laboratory of Molecular Cardiology, Johannes Gutenberg University Medical Center, Mainz, Germany

[4]German Center for Cardiovascular Research (DZHK), Partner Site Rhine-Main, Mainz, Germany

Correspondence:

Dr. Ning Xia or Prof. Dr. Huige Li, Department of Pharmacology, Johannes Gutenberg University Medical Center, Langenbeck Str. 1, 55131 Mainz, Germany. E-Mail: xianing@uni-mainz.de (N.X.); huigeli@uni-mainz.de (H.L.)

Supplementary table S1. Primer list for gene expression studies using qPCR

COX1-F	CCTCTGTACCCAAAGACTGTCC
COX1-R	AAGTGTTGTGCAAAGAAAGCAA
COX2-F	CAGATGCTATCTTTGGGGAGAC
COX2-R	CACCCTTTCACATTATTGCAGA
Cx37-F	CAAGCAGGCGAGAGAGGC
Cx37-R	CGGAAGATGAAGAGCACCGT
Cx40-F	GGAGGAAAGGAAGCAGAAGGCT
Cx40-R	AGACCTTGCCGATGACCGTA
eNOS-F	GGAGGTTCACCGCGTGC
eNOS-R	GACGCTGGTTGCCATAGTGAC
GAPDH-F	TTCTTGTGCAGTGCCAGCC
GAPDH-R	CGTCCGATACGGCCAAATC
iK1-F	TGGCTGAGCACCAAGAG C
iK1-R	TACAGCACCCACTTGCAACC
SK3-F	CACCTTCCCCAAAGCCAACA
SK3-R	ACGAAAACATGGAATCCTTTGAGT
SOD3	GGGACCAAGCCTGTGATCTGT
SOD3	GACCTGGAGATCTGGATGGA
TrpV1-F	TTCACCGAATGGGCCTATGG
TrpV1-R	TGACGGTTAGGGGTCTCACT
TrpV4-F	GCCCATGGATTCGTTGTTCG
TrpV4-R	TGTGGCTGCTTCTCTACGAC

Supplementary table S2. Primer list for chromatin accessibility and ChIP-qPCR studies

COX1-F	TTGGTAAAAAGGGGGCTCAAC
COX1-R	AGTCAACGGTTTACGAGCAT
Cx37-F	GTAGAGGGGACGACCGTGG
Cx37-R	GCAGCTGCGCGCTATTTAAG
SF3-F	CACCACGGTGACAATGTTGG
SK3-R	TCACTCCAGATGGGAGCAGT
TrpV1-F	GAGTATGCCCAGAGCCCATC
TrpV1-R	CAGGCTGCTGTGTGGTAAGA

Supplementary data

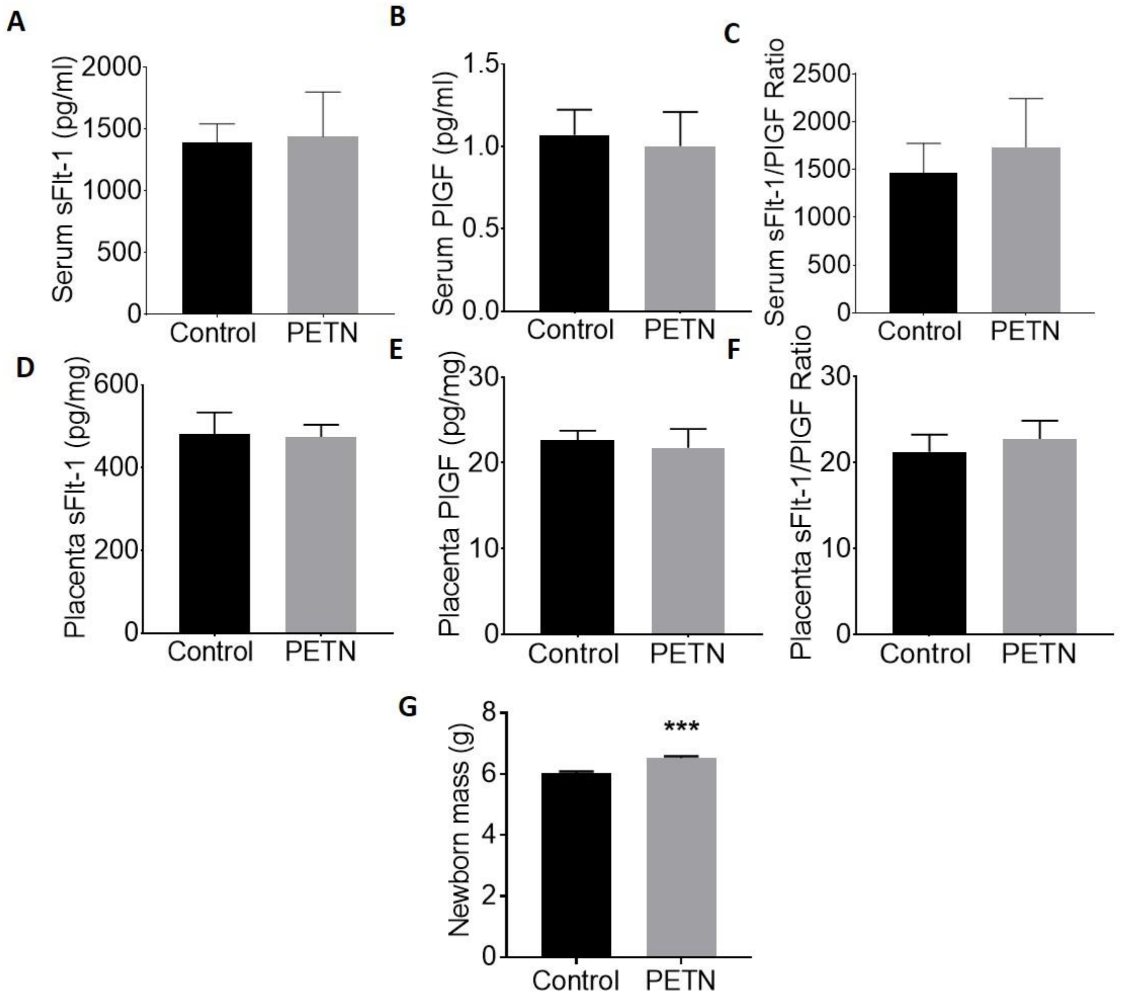

Fig. S1. Maternal PETN treatment of DSSR has no effect on preeclampsia markers. F0 DSSR were treated with or without PETN (50 mg/kg/day) during pregnancy and lactation periods. At the end of pregnancy, serum level of sFlt-1 **(A)** and PlGF **(B)** was measured with commercial ELISA assays and the serum sFlt-1/PlGF ratio was calculated **(C)**. Placenta collected from pregnant DSSR was homogenized and the amount of sFlt-1 **(D)** and PlGF **(E)** was measured with commercial ELISA assays and the placenta sFlt-1/PlGF ratio was calculated **(F).** The newborn weight was measured at day 1 of the infancy. Symbols represents mean ± SEM, n=6. Student’s t test and one-way ANOVA were used for comparison of PETN group with control group. ****P*<0.001, vs control group.

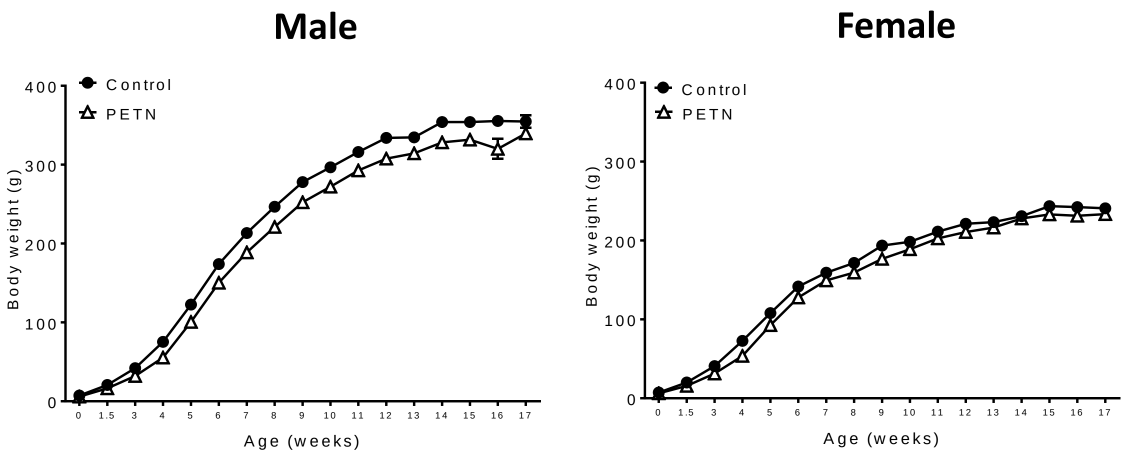

Fig. S2. Maternal PETN treatment has no effect on body weight change in the HSD-fed offspring. F0 DSSR were treated with or without PETN (50 mg/kg/day) during pregnancy and lactation periods. F1 DSSR male and female from all groups received LSD starting at the age of 7 weeks followed by HSD (8% NaCl) starting at the age of 8 weeks. Body weight of male and female F1 DSSR was monitored from birth to the age of 17 weeks. n=3-6.

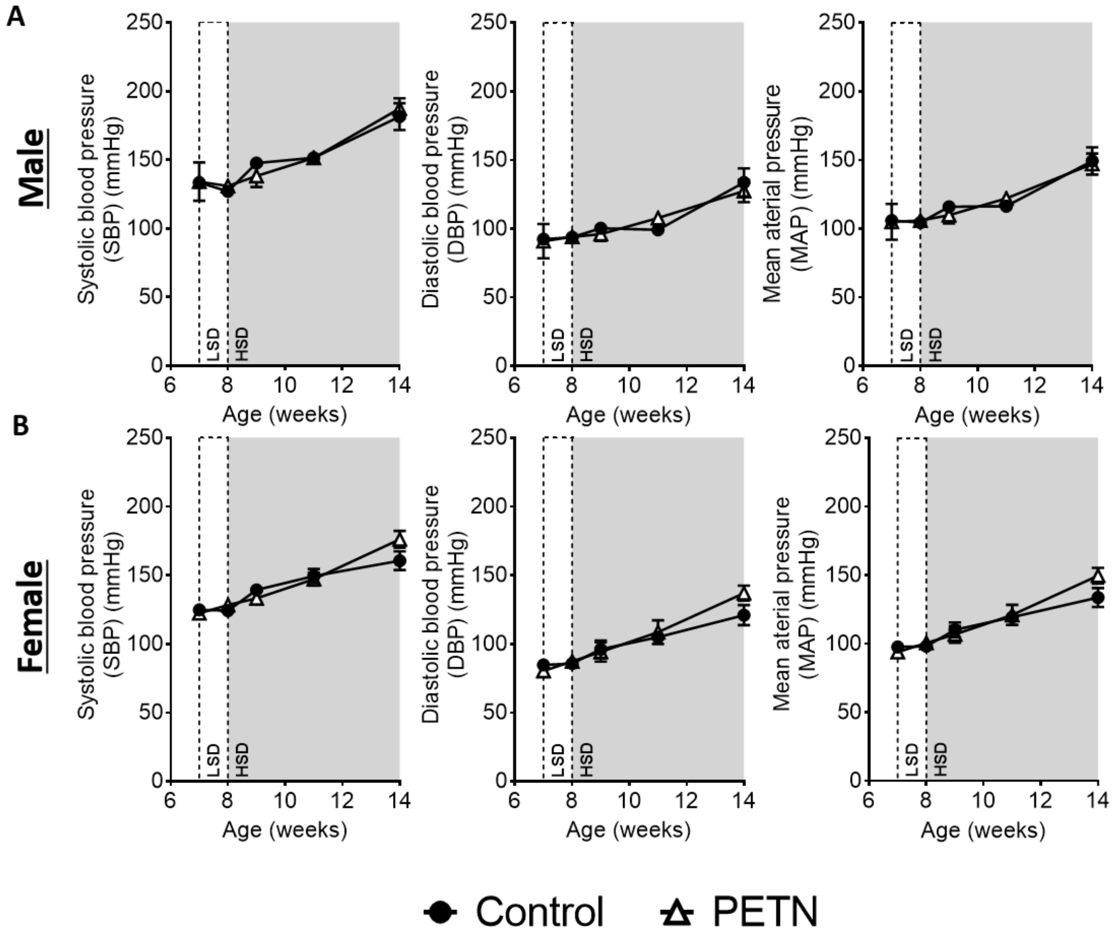

Fig. S3. Maternal PETN treatment has no effect on blood pressure development in the HSD-fed F1 DSSR. F0 DSSR were treated with or without PETN (50 mg/kg/day) during pregnancy and lactation periods. F1 DSSR male and female from all groups received LSD (0.369% NaCl) at the age of 7 week and challenged with HSD (8% NaCl) at the age of 8 weeks. Systolic blood pressure **(SBP)**, diastolic blood pressure **(DBP)** and mean arterial pressure **(MAP)** were measured in the male **(A)** and female **(B)** offspring. Symbols represent mean ± SEM, n=3-6. Student's t test was used for comparison of PETN group with control group at each time point.

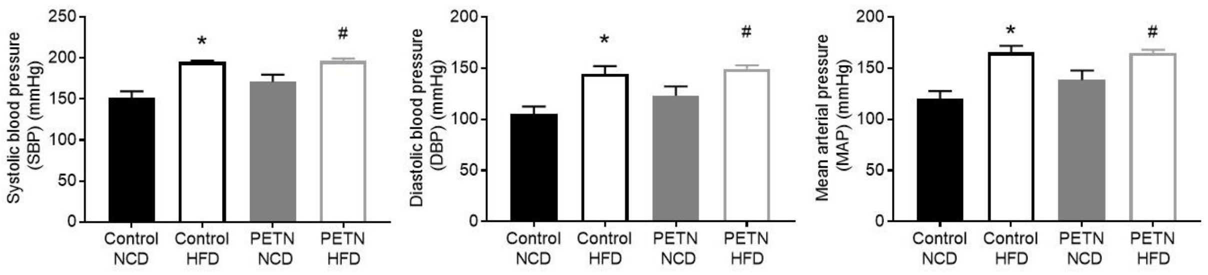

Fig. S4. Maternal PETN treatment has no effect on blood pressure development in HFD-fed F1 female DSSR. F0 DSSR were treated with or without PETN (50 mg/kg/day) during pregnancy and lactation periods. F1 DSSR female offspring received either normal chow (NCD) or high-fat diet (HFD) (45% kcal from fat) starting at the age of 5 weeks. Systolic blood pressure **(SBP)**, diastolic blood pressure **(DBP)** and mean arterial pressure **(MAP)** were measured in the female offspring at age of 30 weeks. Columns represent mean ± SEM, n=3-5. Student's t test was used for comparison. **P*<0.05, vs control-NCD group. #*P*<0.05, vs PETN-NCD group.

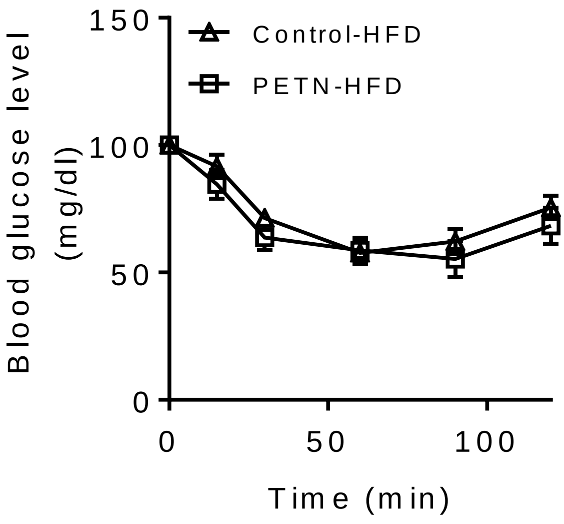

Fig. S5. Effects of maternal PETN treatment on glucose metabolism in 16-weeks HFD-fed offspring. Insulin tolerance test (ITT) was performed in HFD-fed 16-weeks old male DSSR offspring with or without maternal PETN treatment. Data were presented as mean ± SEM, n=3-6.

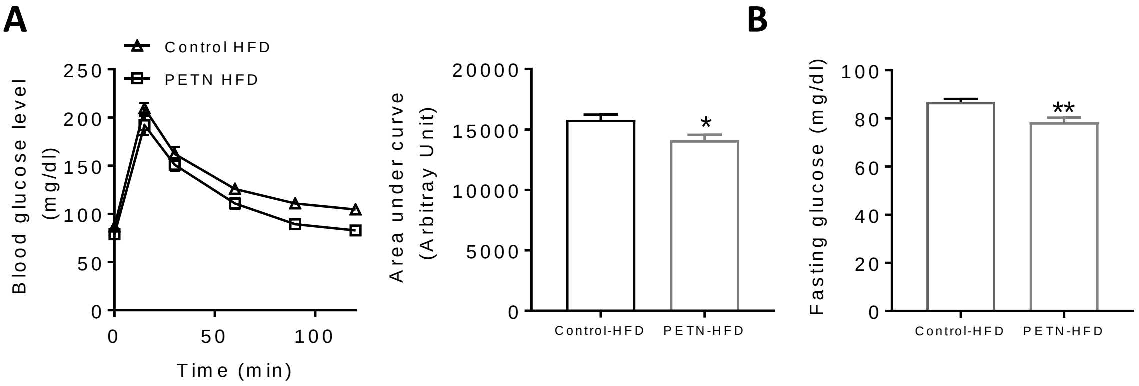

Fig. S6. Effects of maternal PETN treatment on glucose metabolism in HFD-fed offspring. F0 DSSR were treated with or without PETN (50 mg/kg/day) during pregnancy and lactation periods. F1 DSSR male offspring received either normal chow (NCD) or high-fat diet (HFD) (45% kcal from fat) starting at the age of 5 weeks. **(A)** Glucose tolerance test (GTT) was performed in HFD-fed 28-week-old male DSSR and the area under curve was calculated for comparison. **(B)** Glucose level of F1 DSSR after overnight fasting was measured. Data were presented as mean ± SEM, n=8. Student's t test and one-way ANOVA were used for comparison of respective NCD and HFD group. *P<0.05, **P<0.01, vs control-HFD group.

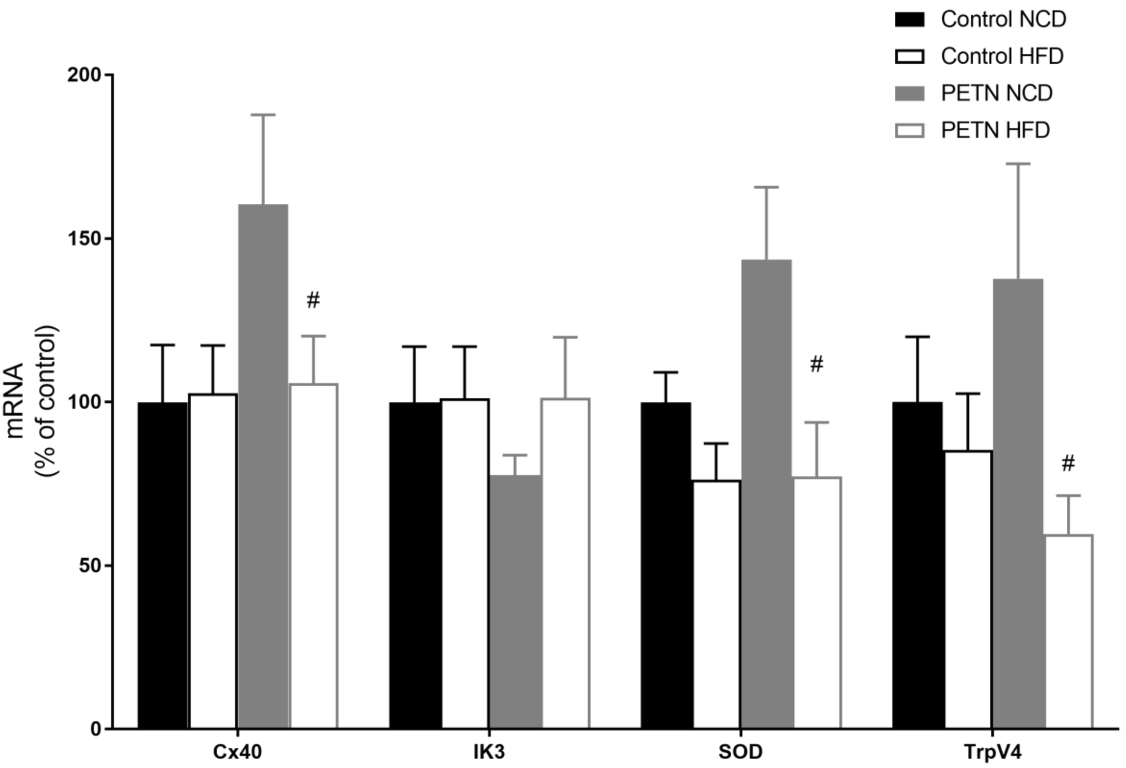

Fig. S7. Effects of maternal PETN treatment on gene expression in aorta of HFD-fed F1 DSSR. F0 DSSR were treated with or without PETN (50 mg/kg/day) during pregnancy and lactation periods. F1 DSSR male offspring received either normal chow (NCD) or high-fat diet (HFD) (45% kcal from fat) starting at the age of 5 weeks. Gene expressions of connexin-40 (Cx40), intermediate conductance Ca^{2+}-activated K^{+} channels 3 (IK3), superoxide dismutase 3 (SOD) and transient receptor potential cation channel subfamily V member 4 (TrpV4) were analyzed in the aorta of the 16-week-old male F1 DSSR with quantitative real-time PCR. Columns represent mean ± SEM. n = 6. Student's t test was used for comparison of respective NCD and HFD group. #*P*<0.05, vs PETN-NCD group.

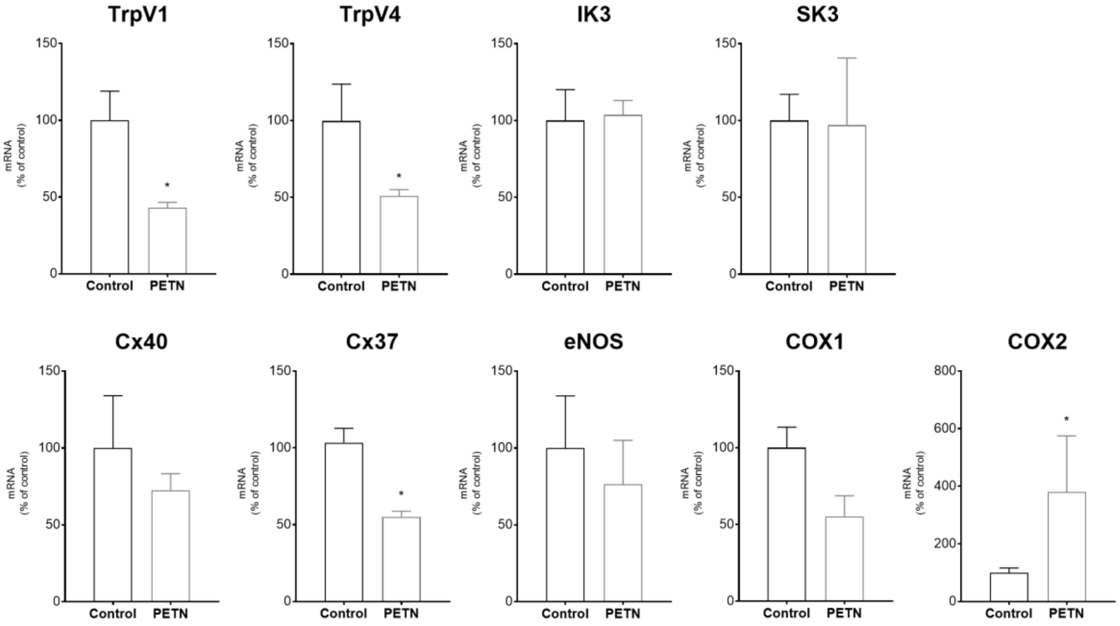

Fig. S8. Effects of maternal PETN treatment on gene expression in mesenteric arteries of HFD-fed F1 DSSR. F0 DSSR were treated with or without PETN (50 mg/kg/day) during pregnancy and lactation periods. F1 DSSR male received high-fat diet (HFD) (45% kcal from fat) starting at the age of 5 weeks. Gene expressions of transient receptor potential cation channel subfamily V member 1 (TrpV1) and 4 (TrpV4), intermediate conductance Ca^{2+}-activated K^+ channels 3 (IK3), small conductance calcium-activated potassium channel 3 (SK3), connexin-40 (Cx40) and connexin-37 (Cx37), endothelial nitric oxide synthetase (eNOS), cyclooxygenase 1 (COX1) and 2 (COX2) were measured in the mesenteric arteries of the HFD-fed 16-week-old male F1 DSSR with quantitative real-time PCR. Columns represent mean ± SEM. n = 3. Student's t test was used for comparison of control and PETN group. *P<0.05, vs control group.
